# Maternal Response to Therapeutic Plasma Exchange in Early Gestation: A Case Series of Thrombotic Microangiopathies and Neurological Disorders

**DOI:** 10.3390/biomedicines14061403

**Published:** 2026-06-22

**Authors:** Onur Karaaslan, Gürcan Türkyılmaz, Latif Hacıoğlu, Çağrı Ateş, Ersin Onat, Erbil Karaman, Hanım Güler Şahin, Ali Doğan

**Affiliations:** 1 Department of Obstetrics and Gynaecology, Faculty of Medicine, Van Yüzüncü Yıl University, 65080 Van, Turkey; dr.latifhacioglu123@hotmail.com (L.H.); ates.cagri@gmail.com (Ç.A.); adar.onat@gmail.com (E.O.); erbil84@gmail.com (E.K.); drsahin@yahoo.com (H.G.Ş.); 2 Department of Obstetrics and Gynaecology, Faculty of Medicine, Lokman Hekim University, 06510 Ankara, Turkey; gurcanturkyilmaz@gmail.com; 3 Department of Haematology, Faculty of Medicine, Van Yüzüncü Yıl University, 65080 Van, Turkey; alidogan@yyu.edu.tr

**Keywords:** therapeutic plasma exchange, thrombotic microangiopathies, pregnancy, neurological disorders

## Abstract

**Background/Objectives:** Therapeutic plasma exchange (TPE) is an extracorporeal treatment used in thrombotic microangiopathies (TMAs) and various autoimmune and neurological disorders. However, data regarding its use during early pregnancy remain limited. This study aimed to evaluate maternal laboratory response and perinatal outcomes in pregnant women who underwent TPE before 26 weeks of gestation. **Methods**: This retrospective case series included 10 pregnant women diagnosed before 26 weeks of gestation who underwent TPE between 2010 and 2023. Clinical and laboratory parameters before and after TPE were compared. **Results**: Indications for TPE included HELLP syndrome (*n* = 4), thrombotic thrombocytopenic purpura (*n* = 3), presumed atypical haemolytic uremic syndrome (*n* = 1), neuromyelitis optica (*n* = 1), and Guillain–Barré syndrome (*n* = 1). The mean gestational age at diagnosis was 22.1 ± 3.1 weeks, and the mean gestational age at delivery was 27.1 ± 6.9 weeks. Five fetuses (50%) died and five (50%) survived to discharge. In patients with TMAs, TPE was associated with significant decreases in LDH, INR, APTT, ALT, AST, and total bilirubin levels, along with a significant increase in platelet count and ADAMTS13 activity (*p* < 0.01). No maternal complications occurred in neurological cases, all of which resulted in term deliveries with healthy neonates. **Conclusions**: In this uncontrolled case series, TPE was associated with rapid maternal clinical and laboratory improvement in selected pregnant women with TMAs, although a causal effect cannot be established from these data. However, perinatal outcomes were primarily determined by gestational age at delivery: all fetal losses occurred before 26 weeks, whereas all infants survived when delivery occurred after 26 weeks. Larger studies are needed to confirm these findings.

## 1. Introduction

Therapeutic plasma exchange (TPE), the most commonly used apheresis procedure, is an extracorporeal treatment that removes soluble components from the plasma. TPE aims to remove circulating pathogenic substances, such as autoantibodies, immune complexes, and ultra-large von Willebrand factor multimers, or to replenish deficient plasma components, such as ADAMTS13. In TPE, the patient’s blood is passed through medical equipment that separates plasma from other blood components, which are subsequently extracted and reinfused with a replacement fluid, such as albumin, saline, or fresh frozen plasma [1]. TPE is widely used in conditions like autoimmune diseases, thrombotic microangiopathies (TMAs) or neurological disorders. American Society for Apheresis (ASFA) guidelines provide clear definitions and good practice recommendations for the different apheresis procedures and their indications. The most recently published version addresses the evidence supporting the use of apheresis to treat more than 80 diseases and 180 indications [2].

Although many ASFA-indicated disorders can occur during pregnancy, limited information exists on TPE use in this population. Some authors suggested that TPE may be conducted safely during pregnancy while warning of potential adverse effects such as hypotension, which can potentially result in lower placental perfusion and fetal oxygenation. Furthermore, significant physiological changes during pregnancy, including increased blood volume, haemodilution, cardiac output, and lower vascular resistance, complicate the TPE technique in pregnant women [3,4].

The rationale for using TPE during pregnancy remains largely empirical rather than evidence-based. The underlying assumption is that removal of pathogenic substances may benefit both mother and fetus. Moreover, treating physicians face a significant barrier to initiating TPE in a pregnant woman, most likely because of the rarity of TPE in pregnancy and a lack of high-quality scientific evidence on its efficacy and safety during pregnancy.

The most common conditions in pregnancy for TPE are TMAs including thrombotic thrombocytopenic purpura (TTP), atypical haemolytic uremic syndrome (aHUS) and HELLP syndrome. Also, Guillain–Barré syndrome, some neuropathies and metabolic diseases may improve after TPE. In the literature, TPE has been used in most cases in the postpartum period in the pregnant population. Data on the use of TPE in the antepartum period, especially in the early weeks of pregnancy, are quite limited.

Most published data on TPE in pregnancy derive from postpartum or third-trimester use, and the antepartum period before fetal viability remains particularly poorly characterised. This distinction is clinically important, because before viability the fetus cannot be salvaged by delivery, and the only way to improve perinatal outcome is to stabilise the mother and prolong the pregnancy. We therefore selected 26 weeks of gestation as our threshold, as it approximates the lower limit of neonatal viability in our setting and separates pregnancies in which delivery offers a realistic chance of neonatal survival from those in which it does not. What remains unknown is whether TPE performed at these very early gestational ages can meaningfully alter maternal disease course and, in turn, perinatal outcome. The present study addresses this gap by focusing specifically on women who underwent TPE before 26 weeks of gestation.

This study aimed to evaluate the maternal clinical and laboratory response to TPE in pregnant women diagnosed before 26 weeks of gestation and to analyse perinatal outcomes according to the gestational age at delivery and underlying disease.

## 2. Materials and Methods

This retrospective case series included all consecutive pregnant women diagnosed before 26 weeks of gestation who underwent therapeutic plasma exchange for any indication at a tertiary referral center between 2010 and 2023. All eligible patients were included. Ethical approval was obtained from the local ethics committee, and written informed consent was obtained from all patients. Clinical data were retrieved from the electronic medical records. Demographic characteristics, including maternal age, BMI, parity, gestational age at diagnosis, gestational age at delivery, birthweight, mode of delivery, number of TPE sessions, and timing of TPE, were recorded.

Laboratory parameters, including haemoglobin, platelet count, LDH, APTT, ALT, AST, fibrinogen, creatinine, total bilirubin, and ADAMTS13 activity, were recorded before and after TPE. For each patient, the value obtained immediately before the first TPE session was used as the pre-treatment value, and the value obtained after completion of TPE was used as the post-treatment value.

Fetal and neonatal outcomes were defined as follows: intrauterine fetal demise (IUFD), neonatal death (with the exact postnatal day), survival at discharge, and survival at 28 days of life. Maternal outcomes included length of intensive care unit (ICU) stay, requirement for renal replacement therapy, neurological recovery status, thrombotic events, disseminated intravascular coagulation (DIC), pulmonary edema, and transfusion requirements.

HELLP syndrome was diagnosed based on the presence of all three criteria according to the Tennessee classification: 1. hemolysis (LDH > 600 U/L, increased total bilirubin, presence of fragmented erythrocytes (schistocytes) in the peripheral smear), 2. elevated liver enzymes (AST > 70 U/L), and 3. low platelet count (<150,000). Furthermore, patients with normal or slightly lower ADAMTS13 levels were excluded from TTP [5]. TTP was diagnosed based on low platelet count (<100,000), in conjunction with an LDH > 2× upper limit of normal and blood film features of microangiopathic haemolytic anaemia. A severely reduced ADAMTS13 activity level (<10%) was diagnostic of TTP [6]. aHUS was associated with schistocytes, elevated lactate dehydrogenase (typically > 600 IU/L), decreased haptoglobin, decreased haemoglobin, and thrombocytopenia (platelet count < 150,000). Complement analysis and genetic testing were not available; therefore, the diagnosis of aHUS was based on clinical and laboratory findings after exclusion of TTP. These lab abnormalities also coincide with one or more of the following: neurological symptoms, acute renal failure, or gastrointestinal symptoms. Normal or slightly reduced ADAMTS13 levels excluded those cases from TTP [7]. In the presumed aHUS case, the diagnosis was favoured over severe HELLP syndrome on the basis of progressive acute kidney injury that dominated the clinical picture and persisted despite delivery, together with relatively preserved liver enzymes and the absence of the marked hepatic involvement that characterises HELLP syndrome; TTP was excluded by a non-severely reduced ADAMTS13 activity level.

The diagnosis of Guillain–Barré syndrome was based on clinical and laboratory criteria including progressive symmetrical weakness that ascends from the lower extremities, along with areflexia, paresthesia or neuropathic pain. A lumbar puncture and electrophysiological examination were performed. Cranial and lumbar MRIs were also utilized [8]. Neuromyelitis optica was diagnosed in the presence of optic neuritis, transverse myelitis and at least one of continuous spinal cord lesions extending more than 3 vertebral segments on MRI or the presence of serum NMO-immunoglobulin G anti-aquaporin4 antibodies [9].

All patients were admitted to the hospital’s intensive care unit (ICU), and supportive care was obtained. TPE indication was determined for each patient by joint decision with the maternal–fetal medicine specialist, haematologist, adult intensive care specialist and neurologist. A central venous catheter was inserted, and TPE was initiated in the intensive care unit either during the antepartum period or within 24–48 h after delivery, according to the clinical condition of the patient. All procedures were performed by membrane filtration using a multiFiltrate system (Fresenius Medical Care, Bad Homburg, Germany). Total blood volume was calculated according to Gilcher’s method, and total plasma volume was derived using the formula TPV = TBV × (1 − haematocrit) [10]. An average of 1.5 plasma volumes was exchanged per session, and the exchanged plasma volume and the volume of replacement fluid were determined individually based on each patient’s weight, height, haematocrit, and clinical and laboratory characteristics. Fresh frozen plasma was the main replacement fluid, and albumin was added when clinically indicated; each unit of fresh frozen plasma and albumin corresponded to 250 mL and 50 mL, respectively. Citrate was used for anticoagulation. The number and frequency of sessions were not fixed but were determined individually according to the clinical and laboratory response, as detailed in the Results.

Vascular access was obtained via central venous catheters inserted into the femoral, jugular, or subclavian veins. During statistical evaluation, tunneled catheters were classified as central venous access, whereas fistulas were considered peripheral vascular access.

For the evaluation of maternal laboratory response, the temporal relationship between delivery and TPE was taken into consideration. In patients who underwent postpartum TPE, laboratory parameters obtained immediately before the first TPE session were used as the pre-treatment values in order to minimise the confounding effect of delivery on biochemical improvement.

Statistical analysis was performed using the Statistical Package for the Social Sciences (SPSS) version 20.0 (IBM Corp., Armonk, NY, USA). Continuous variables were expressed as mean ± standard deviation or median (minimum–maximum) according to the distribution of data, while categorical variables were presented as number and percentage. The normality of the data was assessed using the Shapiro–Wilk test. For comparisons of laboratory parameters before and after TPE, the Wilcoxon signed-rank test was used due to the small sample size and non-normal distribution. A *p* value of <0.05 was considered statistically significant. All laboratory comparisons were performed using values obtained immediately before the first TPE session and after completion of TPE. Because of the small sample size and the exploratory nature of the study, no correction for multiple comparisons was applied; the laboratory analyses should therefore be regarded as hypothesis-generating.

## 3. Results

Ten pregnant women diagnosed before 26 weeks of gestation who underwent TPE for different indications were included in the analysis. Maternal laboratory response and perinatal outcomes were evaluated according to the underlying disease and gestational age at delivery. TPE indications included HELLP syndrome in four cases, TTP in three, presumed aHUS in one patient, neuromyelitis optica in one patient, and Guillain–Barré syndrome in one patient. The mean maternal age was 31.3 ± 4.9 years, the mean gravida was 2.7 ± 1.5, and the mean parity was 1.9 ± 1.1. Mean BMI was 28.5 ± 3.8, and the majority of patients were overweight or obese. The mean gestational age at diagnosis was 22.1 ± 3.1 weeks, and the mean gestational age at delivery was 27.1 ± 6.9 weeks. The mean birthweight was 1120 ± 955 g. Five fetuses (50%) died and the remaining five (50%) survived. Caesarean delivery was performed in seven (70%) women, whereas three (30%) women delivered vaginally. Six (60%) patients underwent one TPE session, three (30%) underwent two sessions, and one (10%) underwent three sessions. Of the 15 TPE sessions performed, three (20%) were antepartum and twelve (80%) were postpartum. The clinical and demographic characteristics of the patients are summarized in Table 1.

The temporal sequence of events differed between the two groups. In the seven TMA patients (HELLP syndrome and TTP), diagnosis was made at a very early gestational age and was rapidly followed by delivery, with TPE initiated postpartum within 24–48 h; in these cases the main maternal complications—cerebral thrombosis in one TTP patient, and disseminated intravascular coagulation and pulmonary oedema in two HELLP patients—occurred in the peripartum and early postpartum period. In contrast, in the two neurological cases and the single presumed aHUS case, TPE was started antepartum after diagnosis, and pregnancy continued for a longer interval, reaching term in both neurological cases. The gestational age at diagnosis, the gestational age at delivery, and the antepartum or postpartum timing of TPE for each case are detailed in Table 2.

All patients with TMAs (HELLP syndrome, TTP, and aHUS) exhibited significant decreases in LDH, INR, APTT, ALT, AST, and total bilirubin concentrations. Conversely, a significant rise in platelet counts and ADAMTS13 levels was observed following TPE therapy (*p* < 0.01). Although haemoglobin levels increased and creatinine levels decreased following TPE, these improvements were not statistically significant. TPE had no significant effect on fibrinogen levels. Laboratory changes before and after TPE treatment in patients with TMAs are shown in Table 3.

Complement analysis and genetic testing for presumed aHUS were not available. The sFlt-1/PlGF ratio was not routinely performed during the study period.

Three cases diagnosed with TTP had severely reduced ADAMTS13 activity levels, and all were delivered before 26 weeks of gestation. None of the fetuses survived beyond 28 days after delivery. In one case, cerebral thrombosis occurred and thrombolytic treatment was required. In all three cases, ADAMTS13 levels increased rapidly after TPE. In four women who underwent TPE due to HELLP syndrome, ADAMTS13 levels were within the normal or mildly reduced range. In two cases, neonatal death occurred due to extreme prematurity, whereas two infants survived beyond 28 days after delivery. In one case, DIC occurred but resolved rapidly after TPE. Pulmonary edema was detected in one patient. In women who underwent TPE for neuromyelitis optica and Guillain–Barré syndrome, no notable maternal complications occurred after TPE. Moreover, these pregnancies progressed uneventfully and resulted in term deliveries of healthy infants. The patient who underwent TPE for aHUS progressed to chronic renal failure and required dialysis. She delivered after 26 weeks of gestation by caesarean section, and the infant survived. Clinical features and maternal and perinatal outcomes of the cases are shown in Table 2.

## 4. Discussion

In this case series of pregnant women diagnosed before 26 weeks of gestation, therapeutic plasma exchange resulted in a rapid and marked improvement in maternal laboratory parameters, particularly in thrombotic microangiopathies. However, this biochemical response did not translate into improved perinatal survival in pregnancies that required delivery at very early gestational ages. In our small cohort, perinatal outcome appeared to be closely associated with gestational age at delivery: all fetal losses occurred before 26 weeks, whereas all pregnancies that could be prolonged beyond this threshold resulted in neonatal survival, irrespective of the magnitude of maternal laboratory recovery. This 26-week threshold was identified post hoc from the observed data rather than defined a priori, and the perfect association seen here should be interpreted with caution given the small sample size and the absence of a validation cohort. Unmeasured factors such as disease severity, the timing of TPE relative to delivery, and placental function are likely to have contributed to perinatal outcome alongside gestational age. By defining pre-treatment laboratory values as those obtained immediately before the first TPE session, especially in patients who underwent postpartum TPE, we aimed to minimise the confounding effect of delivery. Therefore, our findings suggest that the principal clinical role of TPE in early pregnancy is maternal stabilisation, which may enable prolongation of the pregnancy and thereby appears to be an important determinant of fetal survival.

No clinical trials of TPE have been conducted specifically in pregnant women. Its use therefore relies on extrapolation from non-pregnant populations. Furthermore, the use of TPE during pregnancy is mostly empirical, relying on individual case reports in the absence of high-quality studies and straightforward evidence-based guidelines. No pregnancy-specific safety concerns have been reported with TPE compared with its use in the general population. Although hypotension is more commonly reported during TPE in pregnancy than in non-pregnant women, other safety concerns about TPE in pregnancy appear to be favourable. Therefore, TPE procedures have been documented to be used safely during the entire course of pregnancy to treat acute or chronic maternal problems and to minimise fetal death and morbidity, including prolongation of pregnancy to reduce prematurity-associated morbidity [11].

TMA refers to a pattern of variable severity endothelial cell injury that preferentially affects the kidneys, brain and heart. Pregnancy is a high-risk time for the development of different kinds of TMAs. It is diagnosed based on the presence of a low platelet count (<100.000), a haemoglobin level <10 g/dL, LDH > 1.5 upper limit of normal, undetectable serum haptoglobin, negative direct Coombs test and the presence of schistocytes on blood smear or TMA features in kidney or another organ biopsy [12]. Three major syndromes should be sought in pregnancy associated with TMAs including TTP, aHUS and HELLP syndrome.

Precise differentiation between HELLP syndrome, TTP, and presumed aHUS is crucial because management strategies differ, and ADAMTS13 activity plays a central role in this distinction. HELLP syndrome is much more common than TTP and aHUS in pregnancy and the postpartum period. HELLP syndrome is a TMA secondary to increased circulating levels of placenta-derived anti-angiogenic factors such as sFlt-1. TTP is an ADAMTS13-deficient TMA, whereas aHUS is linked to complement dysregulation [13].

HELLP syndrome is a classic form of TMA during pregnancy and the postpartum period. Prompt delivery is the primary treatment for HELLP syndrome. Most cases undergo a rapid recovery after birth and show a notable improvement in platelet count during the initial 48 h of expectant therapy. Occasionally, some cases may not respond to standard supportive care after birth, leading to rapidly worsening clinical and biochemical features with evidence of consumptive coagulopathy and multiorgan failure. In those cases, TMA is critical in the pathophysiology [14]. TPE may involve removing anti-angiogenic factors and increasing complements, thereby resolving TMA and improving recovery in HELLP patients [15]. Moreover, according to the ASFA guidelines, TPE is considered a potentially beneficial therapy for severe cases of postpartum HELLP syndrome. It is classified as a category 3 treatment, which is specifically defined as the efficacy of TPE treatment application remains uncertain, and decisions are made on a case-by-case basis [2]. The remaining indications in our series occupy markedly different positions within the current ASFA framework. Acquired TTP is a Category I, Grade 1A indication, for which TPE is unequivocally first-line therapy, and severe ADAMTS13 deficiency in our three TTP cases is consistent with this recommendation. Guillain–Barré syndrome is likewise a Category I, Grade 1A indication, whereas acute attacks of neuromyelitis optica spectrum disorder are classified as Category II, Grade 1B. For aHUS, ASFA assigns TPE a Category I recommendation when an anti-complement factor H antibody is present and a Category III recommendation for complement gene mutations; because complement and genetic testing were unavailable, our presumed aHUS case could not be formally placed within either subcategory. Our practice was therefore broadly concordant with current ASFA recommendations for the TTP, Guillain–Barré, and neuromyelitis optica indications, while TPE for HELLP syndrome and presumed aHUS was applied on the individualised, case-by-case basis that the guidelines explicitly endorse for these conditions. In recent years, several research papers were published on the role of TPE in HELLP syndrome. Erkut et al. evaluated 47 class-1 HELLP syndrome and found that a dramatic clinical and laboratory improvement occurred after TPE. They suggested that postpartum use of TPE within 24 h is an effective treatment option for class-1 HELLP syndrome [16]. Chowdhry et al. investigated nine cases who underwent TPE after delivery with severe HELLP syndrome and showed that postpartum, early TPE therapy improves clinical outcomes and resolves laboratory problems [17]. In our cohort, we conducted TPE for HELLP syndrome in four cases and maternal clinical and laboratory recovery was achieved rapidly after TPE in all cases.

The diagnosis of acute TTP during pregnancy is based on the same criteria as in the non-pregnant condition. TTP may appear in the first trimester, although the majority of cases occur in the third trimester or puerperium. Clinical characteristics may be similar to those of HELLP syndrome or aHUS. Clinical deterioration and persistent severe thrombocytopenia 48–72 h after birth should raise the possibility of TTP. A severely reduced ADAMTS13 activity level is the cornerstone for distinguishing it from other causes of TMAs [18]. Pregnancy-related TTP is rare and severe ADAMTS13 deficiency affects an estimated 1/200,000 pregnancies and the majority of cases are congenital TTP [19]. The delivery decision depends on maternal safety, fetal maturity and well-being and requires a multidisciplinary approach. In cases of TTP, delivery is not curative solely and TPE is the primary treatment approach. Perinatal outcomes are poor in cases with TTP in pregnancy. Fujimura et al. evaluated 15 women diagnosed with congenital TTP in the second trimester and showed that, of 15 pregnancies, eight babies were stillborn or died soon after birth, and the remaining seven were all premature except one, who was delivered vaginally following maternal plasma infusions initiated at 8 weeks of gestation [20]. Finally in a large cohort of acute TTP in pregnancy within the United Kingdom, involving 35 women and nearly 100 pregnancies, two-thirds of all cases were late-onset congenital TTP. The presentation was primarily in the third trimester or immediately after delivery. There was a 40% fetal loss rate prediagnosis of TTP, almost exclusively in the second trimester, but 100% fetal survival with TPE [21]. In our cohort, three cases were treated with TPE due to TTP and in all cases, pregnancies resulted in perinatal death. Although TPE improved laboratory findings and increased ADAMTS13 activity levels significantly, in one woman, cerebral thrombus occurred after delivery and thrombolytic treatment was required.

In our series, although TPE resulted in a rapid increase in ADAMTS13 activity and improvement in laboratory parameters in all patients with TTP, perinatal outcomes were poor. This finding is most likely related to the very early gestational age at delivery rather than the lack of maternal response to treatment. Therefore, while TPE appears to be effective in stabilising maternal disease, fetal prognosis remains largely dependent on gestational age at the time of diagnosis and delivery.

Guillain–Barré syndrome constitutes a heterogeneous collection of immune-mediated peripheral neuropathies, typically affecting the myelin sheath of nerve fibres and exhibiting a variety of clinical manifestations. Guillain–Barré syndrome occurs rarely in pregnancy with an estimated incidence rate of 1–2/100,000 [22]. TPE and IVIG are the main treatment modalities that achieve complete recovery in 80% of patients particularly within the first two weeks after the onset of the disease [23]. There are a few case reports in the literature investigating the course of Guillain–Barré syndrome in pregnancy. Tulbure et al. demonstrated a woman with Guillain–Barré syndrome in pregnancy at 33 weeks of gestation treated with TPE and IVIG in pregnancy. The patient recovered following treatment and delivered a healthy infant [24]. In our case series, one woman was treated with TPE due to Guillain–Barré syndrome and achieved complete recovery and delivered a healthy baby at term.

Neuromyelitis optica or Devic syndrome is a recurrent antibody-mediated inflammatory disorder characterised by the clinical presentation of optic neuritis and transverse myelitis. Although there are a few case series of neuromyelitis optica in pregnancy in the literature, there is evidence that pregnancy may be associated with disease exacerbation, relapse, and adverse maternal and fetal outcomes [25]. Wuebbolt et al. evaluated 13 pregnancies in women with neuromyelitis optica who were treated with a combination of steroids, immunosuppressants, and TPE. They showed that more than 80% of women delivered healthy babies [26]. In our case series, one mother was treated with TPE owing to neuromyelitis optica and delivered a healthy baby at term. In both neurological cases, intravenous immunoglobulin was used as the initial treatment, consistent with its established first-line role in Guillain–Barré syndrome and in acute attacks of neuromyelitis optica spectrum disorder. TPE was initiated only after an inadequate clinical response to IVIG, as a second-line option intended to remove pathogenic antibodies directly. This sequence reflects routine clinical practice rather than any specific pregnancy-related contraindication to IVIG, and both pregnancies subsequently progressed to term with healthy neonates. As these represent only two patients, however, they are presented as descriptive observations and should not be taken to indicate a general efficacy or safety of TPE for neurological disorders in pregnancy.

This study has several limitations. First, its retrospective design and small sample size limit the generalisability of the findings. Second, both antepartum and postpartum TPE sessions were included in the same analysis. Because seven of the ten patients received TPE only after delivery, the laboratory improvements observed in these cases, particularly in HELLP syndrome, cannot be confidently attributed to TPE rather than to the natural postpartum resolution of the underlying pregnancy-specific condition. Defining the pre-TPE values as those obtained immediately before the first session only partially mitigates this problem, and delivery itself therefore remains an important confounder in the postpartum subgroup. Third, complement and genetic testing were not available, and the sFlt-1/PlGF ratio was not measured during the study period; the diagnosis of aHUS therefore rested on clinical and laboratory findings after exclusion of TTP, and some misclassification between HELLP syndrome and complement-mediated TMA is possible. Finally, the number of TPE sessions performed in our TTP patients was lower than the standard of care for acquired TTP, which requires daily exchange until platelet count and ADAMTS13 activity recover. In our series, all three TTP patients were diagnosed at very early gestational ages and were delivered shortly afterwards, with fetal loss before 26 weeks, so the planned course of TPE could not be completed as it normally would be. The poor perinatal outcomes in these cases, despite rapid laboratory improvement, are therefore more likely to reflect the extremely early gestational age at presentation and delivery, together with a treatment course shorter than current standards recommend, than a true failure of TPE; the occurrence of cerebral thrombosis after only two sessions in one patient is consistent with this interpretation. In addition, multiple laboratory parameters were compared in a small number of TMA patients without correction for multiple testing, which increases the risk of type I error; the statistically significant changes shown in Table 3 should therefore be interpreted as exploratory rather than confirmatory. Despite these limitations, the inclusion of all consecutive early gestational cases, the systematic assessment of ADAMTS13 activity in TMA patients, and the detailed characterisation of maternal and perinatal outcomes represent important strengths of this study.

## 5. Conclusions

In pregnant women diagnosed before 26 weeks of gestation, therapeutic plasma exchange was associated with rapid maternal laboratory and clinical improvement, particularly in thrombotic microangiopathies; however, as this was a retrospective, uncontrolled case series, this improvement cannot be attributed to TPE with certainty. However, our findings demonstrate that fetal survival is primarily determined by the ability to prolong pregnancy rather than by the magnitude of maternal biochemical response. These results underline the critical importance of early recognition and accurate differentiation of TMAs using ADAMTS13 activity and complement assessment. They also highlight the value of timely multidisciplinary management aimed at pregnancy prolongation. In practical terms, when a TMA presents before 26 weeks of gestation, aggressive maternal stabilisation with TPE should be pursued not for the sake of biochemical normalisation itself, but to gain sufficient time to reach a gestational age compatible with neonatal survival. The two neurological cases should be interpreted separately from the TMA group. In both, pregnancy continued to term with favourable maternal and neonatal outcomes, reflecting the absence of a delivery-driven disease course; however, with only two patients these observations are descriptive and do not allow any general conclusion about the efficacy or safety of TPE for neurological disorders in pregnancy. Overall, the small size and clinical heterogeneity of our cohort preclude strong conclusions about TPE as a single unified intervention, and the TMA and neurological indications are best considered separately. Future prospective multicentre studies are required to define the optimal timing, indications and treatment strategies that may maximise both maternal stabilisation and gestational prolongation in early pregnancy.

## Figures and Tables

**Table 1:** Demographic and clinical characteristics of patients (*n* = 10).

Patient’s Characteristics (*n*: 10)	Mean ± SD
Age (years)	31.3 ± 4.9
Gravida	2.7 ± 1.5
Parity	1.9 ± 1.1
BMI (kg/m^2^)	28.5 ± 3.8
GA at diagnosis (weeks)	22.1 ± 3.1
GA at delivery (weeks)	27.1 ± 6.9
Birthweight (gram)	1120 ± 955
Perinatal outcome (*n*, %)	Alive: 5 (50%) Death: 5 (50%)
Preeclampsia/eclampsia history (*n*, %)	Yes: 4 (40%) No: 6 (60%)
Mode of delivery (*n*, %)	Caesarean: 7 (70%) Vaginal delivery: 3 (30%)
TPE number (*n*, %)	1: 6 (60%) 2: 3 (30%) 3: 1(10%)
TPE timing (*n*, %)	Antepartum: 3 (20%) Postpartum: 12 (80%)

(BMI: Body mass index; GA: Gestational age; TPE: Therapeutic plasma exchange).

**Table 2:** Clinical features, maternal and perinatal outcomes of cases (*n*: 10). Perinatal outcome was closely related to gestational age at delivery, with all fetal losses occurring before 26 weeks of gestation.

Case No	Indication	TPE Timing	TPE Number	ADAMTS13 Level (%)	GA at Diagnosis (Weeks)	GA at Delivery (Weeks)	Mode of Delivery	Birthweight (Gram)	Survival at 28 Days (Yes/No)	Maternal Complication
1	TTP	Postpartum	2	7.9	20 + 2	20 + 3	Vaginal	400	No	None
2	TTP	Postpartum	2	6.2	25 + 4	25 + 4	Cesarean	690	No	CT
3	TTP	Postpartum	3	5.1	24 + 4	24 + 5	Cesarean	550	No	None
4	HELLP	Postpartum	2	22.5	23 + 5	24	Cesarean	500	No	DIC
5	HELLP	Postpartum	1	30.2	25 + 1	25 + 3	Cesarean	740	Yes	PO
6	HELLP	Postpartum	1	39.8	22 + 6	23	Vaginal	490	No	None
7	HELLP	Postpartum	1	29.7	26	26	Cesarean	880	Yes	None
8	NO	Antepartum	1	N/A	18 + 4	37 + 4	Vaginal	3100	Yes	None
9	GB	Antepartum	1	N/A	17 + 5	38 + 6	Cesarean	3450	Yes	None
10	aHUS	Antepartum	1	46.5	25 + 2	26 + 2	Cesarean	1010	Yes	RI

TPE: therapeutic plasma exchange; ADAMTS13: a disintegrin and metalloproteinase with a thrombospondin type 1 motif, member 13; GA: gestational age; TTP: thrombotic thrombocytopenic purpura; HELLP: haemolysis, elevated liver enzymes, low platelets; aHUS: atypical haemolytic uraemic syndrome; NO: neuromyelitis optica; GB: Guillain–Barré; CT: cerebral thrombosis; DIC: disseminated intravascular coagulation; PO: pulmonary oedema; RI: renal insufficiency; N/A: not applicable.

**Table 3:** Laboratory changes pattern before and after TPE in cases with TMAs (HELLP syndrome, TTP, HUS) (*n*: 8).

Laboratory Parameters	Before TPE Median (Min–Max)	After TPE Median (Min–Max)	*p* Values
Haemoglobin (g/dL)	7.3 (6–12)	9.0 (6.2–11.4)	0.610
Platelets (×10^3^/µL)	30 (4–141)	256 (130–318)	0.008 **
LDH (U/L)	1562 (234–8426)	318 (134–1876)	0.009 **
INR	1.36 (1.19–1.72)	1.10 (0.95–1.25)	0.012 *
APTT (seconds)	31 (17–50)	19 (13–29)	0.015 *
ALT (U/L)	416 (12–1593)	30 (6–67)	0.009 **
AST (U/L)	426 (13–3133)	39 (14–64)	0.007 **
Fibrinogen (mg/dL)	243 (32–385)	258 (76–507)	0.575
Creatinine (mg/dL)	1.2 (0.3–2.1)	1.25 (0.3–2.2)	0.919
Total bilirubin (mg/dL)	14 (1–37)	2.1 (1.2–8.3)	0.007 **
ADAMTS-13 level (%)	22.3 (9.5–33.8)	49.1 (21.5–84.6)	0.005 **

Data are presented as median (min–max). *p* values were calculated using the Wilcoxon signed-rank test. *p* < 0.05 * *p* < 0.01 **. TMA: thrombotic microangiopathy; LDH: lactate dehydrogenase; INR: international normalised ratio; APTT: activated partial thromboplastin time; ALT: alanine aminotransferase; AST: aspartate aminotransferase; ADAMTS-13: a disintegrin and metalloproteinase with a thrombospondin type 1 motif, member 13; TPE: therapeutic plasma exchange.

## Data Availability

The datasets generated and/or analysed during the current study are not publicly available due to patient confidentiality and the small number of cases, which may compromise anonymity. However, the data are available from the corresponding author on reasonable request.

## References

[1] Colpo A., Marson P., Pavanello F., Tison T., Gervasi M.T., Zambon A., Ruffatti A., De Silvestro G., Hoxha A. (2019). Therapeutic apheresis during pregnancy: A single centre experience. Transfus. Apher. Sci..

[2] Connelly-Smith L., Alquist C.R., Aqui N.A., Hofmann J.C., Klingel R., Onwuemene O.A., Patriquin C.J., Pham H.P., Sanchez A.P., Schneiderman J. (2023). Guidelines on the Use of Therapeutic Apheresis in Clinical Practice—Evidence-Based Approach from the Writing Committee of the American Society for Apheresis: The Ninth Special Issue. J. Clin. Apher..

[3] Marson P., Gervasi M.T., Tison T., Colpo A., De Silvestro G. (2015). Therapeutic apheresis in pregnancy: General considerations and current practice. Transfus. Apher. Sci..

[4] Perrone G., Brunelli R., Marcoccia E., Zannini I., Candelieri M., Gozzer M., Stefanutti C. (2016). Therapeutic Apheresis in Pregnancy: Three Differential Indications With Positive Maternal and Fetal Outcome. Ther. Apher. Dial..

[5] Wallace K., Harris S., Addison A., Bean C. (2018). HELLP Syndrome: Pathophysiology and Current Therapies. Curr. Pharm. Biotechnol..

[6] Scully M. (2021). Congenital TTP: Next stop, acuity and therapy. Blood.

[7] Jokiranta T.S. (2017). HUS and atypical HUS. Blood.

[8] Murthy J.M.K. (2017). Guillain–Barré syndrome and variants: Antiganglioside antibodies. Neurol. India.

[9] Costello F. (2022). Neuromyelitis Optica Spectrum Disorders. Continuum.

[10] Shelat S.G. (2010). Practical considerations for planning a therapeutic apheresis procedure. Am. J. Med..

[11] Wind M., Gaasbeek A.G.A., Oosten L.E.M., Rabelink T.J., van Lith J.M.M., Sueters M., Teng Y.K.O. (2021). Therapeutic plasma exchange in pregnancy: A literature review. Eur. J. Obstet. Gynecol. Reprod. Biol..

[12] Fakhouri F., Scully M., Provôt F., Blasco M., Coppo P., Noris M., Paizis K., Kavanagh D., Pène F., Quezada S. (2020). Management of thrombotic microangiopathy in pregnancy and postpartum: Report from an international working group. Blood.

[13] George J.N., Nester C.M. (2014). Syndromes of thrombotic microangiopathy. N. Engl. J. Med..

[14] Haram K., Svendsen E., Abildgaard U. (2009). The HELLP syndrome: Clinical issues and management. A review. BMC Pregnancy Childbirth.

[15] Baxter J.K., Weinstein L. (2004). HELLP syndrome: The state of the art. Obstet. Gynecol. Surv..

[16] Erkurt M.A., Sarici A., Kuku I., Berber I., Kaya E., Bicim S., Karaman S., Ozgul M. (2021). The effect of therapeutic plasma exchange on management of HELLP syndrome: The report of 47 patients. Transfus. Apher. Sci..

[17] Chowdhry M., Agrawal S., Gajulapalli S.P., Thakur U.K. (2022). Therapeutic plasma exchange in HELLP syndrome: A life saviour. Asian J. Transfus. Sci..

[18] Scully M. (2016). Thrombotic Thrombocytopenic Purpura and Atypical Hemolytic Uremic Syndrome Microangiopathy in Pregnancy. Semin. Thromb. Hemost..

[19] Thomas M.R., Robinson S., Scully M.A. (2016). How we manage thrombotic microangiopathies in pregnancy. Br. J. Haematol..

[20] Fujimura Y., Matsumoto M., Kokame K., Isonishi A., Soejima K., Akiyama N., Tomiyama J., Natori K., Kuranishi Y., Imamura Y. (2009). Pregnancy-induced thrombocytopenia and TTP, and the risk of fetal death, in Upshaw-Schulman syndrome: A series of 15 pregnancies in 9 genotyped patients. Br. J. Haematol..

[21] Scully M., Rayment R., Clark A., Westwood J.P., Cranfield T., Gooding R., Bagot C.N., Taylor A., Sankar V., Gale D. (2023). A British Society for Haematology Guideline: Diagnosis and management of thrombotic thrombocytopenic purpura and thrombotic microangiopathies. Br. J. Haematol..

[22] Fokke C., van den Berg B., Drenthen J., Walgaard C., van Doorn P.A., Jacobs B.C. (2014). Diagnosis of Guillain-Barré syndrome and validation of Brighton criteria. Brain.

[23] Chan L.Y., Tsui M.H., Leung T.N. (2004). Guillain-Barré syndrome in pregnancy. Acta Obstet. Gynecol. Scand..

[24] Iliadi-Tulbure C., Cemortan M., Jubirca S., Cospormac V., Bubulici C., Vicol M.M. (2024). Guillain-Barré syndrome in pregnancy: A case report and review of the literature. AJOG Glob. Rep..

[25] D’Souza R., Wuebbolt D., Andrejevic K., Ashraf R., Nguyen V., Zaffar N., Rotstein D., Wyne A. (2020). Pregnancy and Neuromyelitis Optica Spectrum Disorder—Reciprocal Effects and Practical Recommendations: A Systematic Review. Front. Neurol..

[26] Wuebbolt D., Nguyen V., D’Souza R., Wyne A. (2018). Devic syndrome and pregnancy: A case series. Obstet. Med..

